# The effect of haze pollution on insurance development: Evidence from 268 prefecture-level cities in China

**DOI:** 10.1371/journal.pone.0267830

**Published:** 2022-04-28

**Authors:** Yonglian Chang, Yingjun Huang, Manman Li, Zhengmin Duan

**Affiliations:** 1 School of Economics and Business Administration, Chongqing University, Chongqing, China; 2 Department of Statistics and Actuarial Science, College of Mathematics and Statistics, Chongqing University, Chongqing, China; Sun Yat-Sen University, CHINA

## Abstract

The relationship between haze pollution and insurance development is investigated based on the concentration of PM_2.5_ of 268 Chinese cities during 2009~2018. Subsequently, the effect of haze pollution on the development of insurance and the underlying mechanisms are also explored. The regional governance of haze pollution and its impact on insurance development is estimated by using a unified framework of two-stage least squares. The machine learning method-elastic network is adopted to filter the control variables and avoid multi-collinearity. The results show that haze pollution has an adverse effect on the insurance development through two important underlying mechanisms, residents’ emotions and economic development. Haze pollution affects residents’ emotions, and the impact coefficient is approximately equal to -0.18, which further inhibits residents’ participation in insurance. Moreover, pollution restricts residents’ budgets by hindering economic development, the impact coefficient is about -0.07, thus, the development of insurance is suppressed. These two negative effects exhibit regional variations, which gradually attenuate from eastern, western to the Chinese central region. The regional governance has a positive effect on haze pollution with the coefficient of -0.07, while impact coefficient of haze pollution on insurance development decreases to -0.02. The policy implication is that government supervision can formulate reasonable environmental and insurance policies based on the heterogeneity of regional development to alleviate haze pollution and promote insurance development.

## 1. Introduction

**Fig 1:**
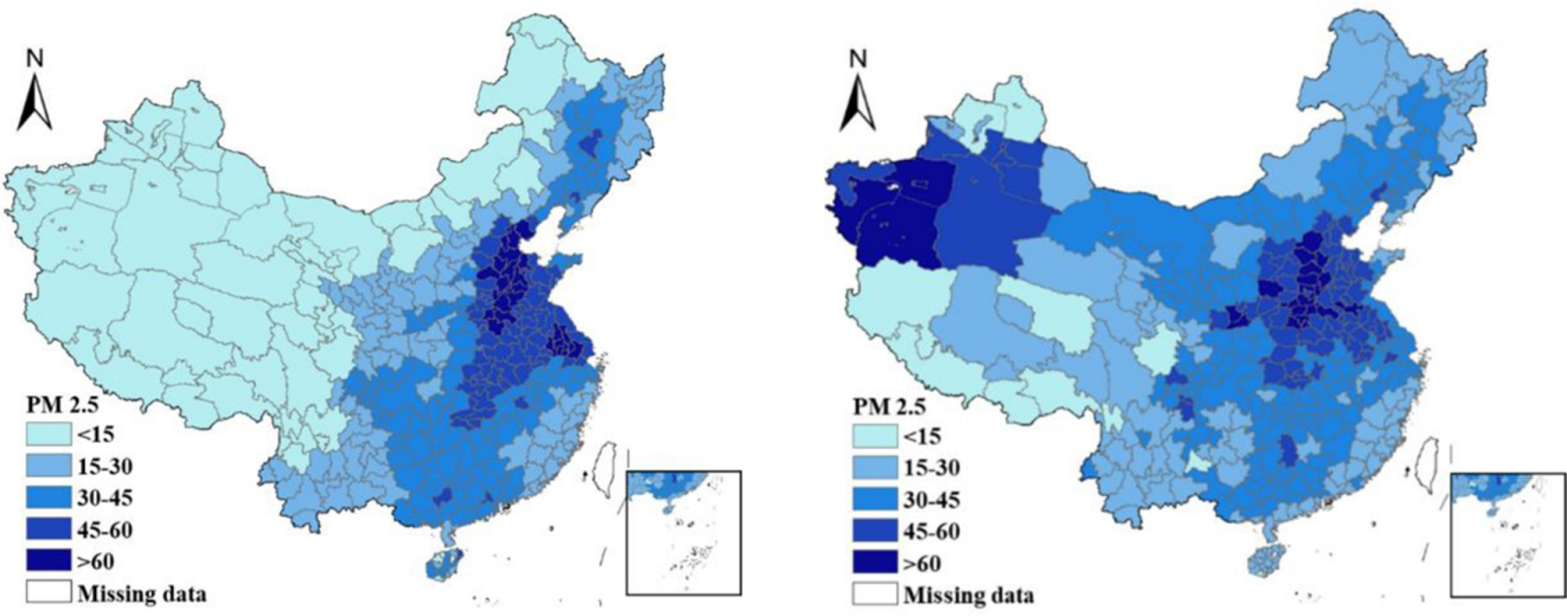
PM_2.5_ concentration of China.

Accompanied with the rapid development of economies, China is suffering from several ensuing issues, which includes population explosion, resource overconsumption, and ecological destruction, especially the environment problem-haze pollution [1]. Although the Chinese government has made great efforts and promulgated a series of environmental regulations to reduce and control the environment problem since 2013 [2], the haze pollution in China is still severe [3] as shown in Fig 1, the haze pollution eastern region is more serious comparing to the central and western regions.

Haze pollution (PM_2.5_) can enter the human body through multiple channels and harms the physical and mental health of residents [3]. The negative effects on the human include morbidity, mortality, cognitive performance [4] and life expectancy [5]. Haze pollution not only threatens people’s health, but also stimulates the need for basic medical insurance from the perspective of avoidance behavior [6]. Few literatures has explored the effect of haze pollution on the insurance, especially the insurance sales of residents’ health [7]. For example, Chang et al. [7] found that a one standard deviation increase in daily air pollution resulted in an increase in commercial health insurance sales for that day, which provided a basic theoretical foundation for following researches. Pi et al. [8] found air pollution show a significantly negative correlation with basic medical insurance, the more severe the haze pollution, the less medical insurance was sold.

The above studies only focused on environment effect on the insurance sales of residents’ health, its impact on the overall insurance market over time has not been reported, and the underlying mechanism of effect of haze pollution on the insurance development has not been explained well.

**Fig 2:**
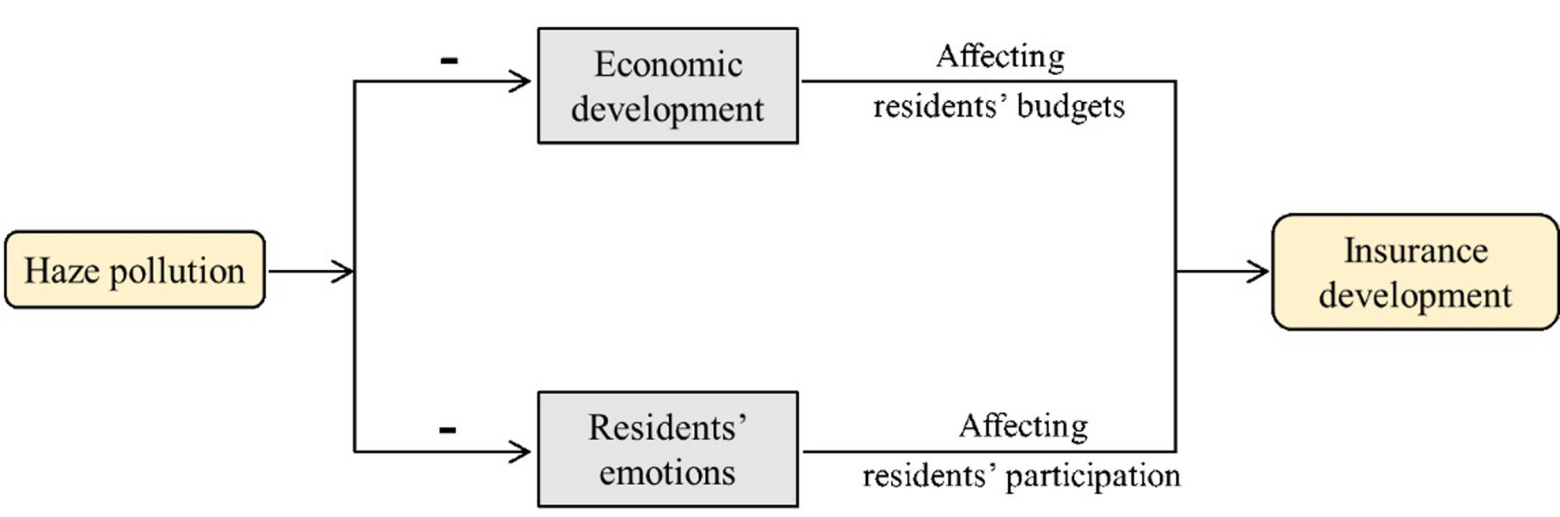
The transmission mechanisms of the effect of haze pollution on insurance development.

Some prior literatures have been discussed the underlying mechanism [5, 9]. Haze pollution have two indirect effects, economic development and residents’ emotion, on insurance development, these two indirect effects are as the bridge to indicate the relationship between the haze pollution and insurance development. Firstly, haze pollution can seriously hindered economic development [9, 10], which will affect residents’ income [11] and affect household budgets for insurance cost [12]. In fact, development degree of the insurance market is determined by the economic level [13–16], which is more evident in developing countries [17]. This is because the high-quality development of the economy will increase residents’ income and budget, which will causes the increasing demand for insurance [18]. Secondly, the residents’ expenditure behavior is also affected by haze pollution [19], which is due to the haze pollution seriously affected the emotions of residents [20] and their decision-making [21, 22]. Therefore, haze pollution will affect residents’ decision for purchasing insurance by affecting residents’ emotions. To sum up, this paper uses economic development and residents’ emotions as two underlying mechanisms to explore the impact of haze pollution on insurance development. The above two mechanisms are shown in Fig 2.

**Fig 3:**
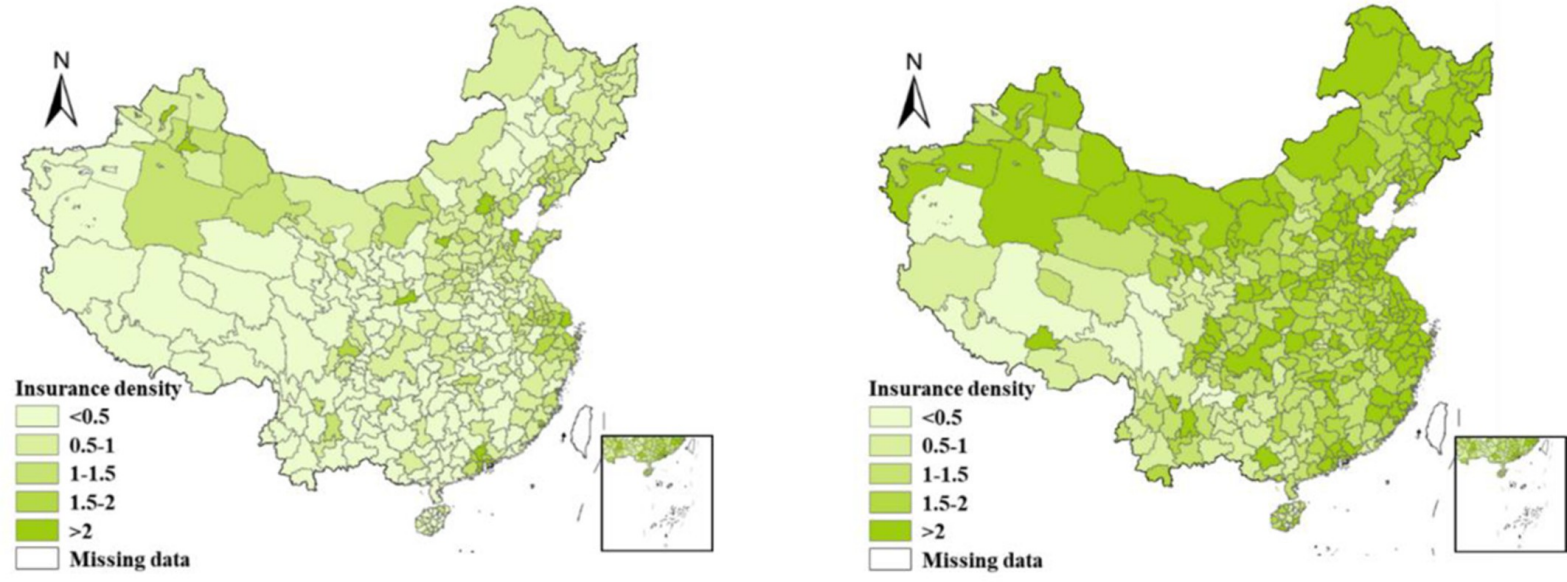
Insurance density of China.

The insurance market in China has rapidly developed in recent years (Fig 3). The above researches provided the rich theoretical basis and unique scientific research perspective. As a trivial contribution, this paper explores the underlying mechanisms of the effect of haze pollution on Chinese insurance market. This paper firstly investigates the relationship between haze pollution and insurance development, machine learning method-elastic network to select the control variables, which is used to avoid multi-collinearity. Then, the effect of two underlying mechanisms, economic development and the residents’ emotions, on Chinese insurance development are analyzed in detail. Thirdly, this paper considers the spillover effect of haze pollution and constructs an air flow coefficient. Finally, the effects of regional environmental governance on haze reduction and insurance development under the unified framework of two-stage least squares estimation are also discussed.

The structure of this study is as follows. Section 2 is the research methodology and using data. Discussion and analysis of the results are presented in Section 3. The instrumental variables regression is adopted in Section 4 to investigate regional governance and insurance development. Section 5 provides conclusions and policy implications.

## 2. Data description and research methodology

### 2.1. Data description

This research examines the influence of haze pollution on the development of Chinese insurance, evaluates impact of regional environment governance. The using data are mainly collected from the two government’s reports “Chinese Insurance Yearbook” and “Chinese City Statistical Yearbook” during 2009–2018 for 268 prefecture-level cities (http://www.stats.gov.cn/tjsj/ndsj/).

The following benchmark regression model is used:

$$Preincome_{i,j}=\alpha_{0}+\alpha_{1}PM_{2.5}{}_{i,j}+\alpha_{2}X_{i,j}+\varepsilon_{i,j}$$ (1)

where *i*, *j* are city, year. *Preincome* denotes premium income, and *PM*_*2*.*5i*,*j*_ is the concentration of PM_2.5_. *X*_*i*,*j*_ indicates the characteristic variables of the city, and *α*_1_ is the core parameter. After the characteristic variables are controlled, if *α*_1_< 0, haze pollution hinders insurance development; otherwise, *α*_1_< 0 indicating that haze pollution will promote insurance development. *ε*_*i*,*j*_ is random error.

This article selects PM_2.5_ concentration as an indicator for haze pollution, the source of the PM_2.5_ is same as study [3], which is collected from Columbia University’s Socioeconomic Data and Application Center, and the ERA-INTERIM raster meteorological data released by the European Center for Medium-Term Weather Forecast (ECMWF). ArcGIS is used to extract the data from the annual PM_2.5_ density map.

### 2.2. Variable selection

This paper collects a set of urban characteristic variables, and the selection principles of the variables are as follows: (1) Refer to the variables used in previous studies (2) Qualitatively assess the impact of each variable on premium income. Finally, 17 original variables are selected.

Due to the diversity and multi-collinearity of the variables, simplicity is particularly important. The elastic network method is adopted to select the control variables, which not only avoids multi-collinearity, but also obtains the independent variables that highly correlate with dependent variables [23].

The dataset of this paper has *N* (*N* = 2680) observations with *p* (*p* = 17) predictors. The premium income *y*_i_ is standardized as following,

$$\sum_{i=1}^{N}y_{i}=0,\;\sum_{i=1}^{N}x_{ij}=0,\;\sum_{i=1}^{N}x_{ij}^{2}=1,\;j=1,\;2\ldots 17,N=2680$$ (2)

Zou and Hastie [23] defines the elastic net criterion by any fixed non-negative *λ*_1_ and *λ*_2_

$$L(\lambda_{1},\lambda_{2},\beta )={\left|y-X\beta \right|}^{2}+\lambda_{2}{\left|\beta \right|}^{2}+\lambda_{1}{\left|\beta \right|}_{1}$$ (3)

$$\hat{\beta }=\underset{\beta }{\arg\min}\{L(\lambda_{1},\lambda_{2},\beta )\}.$$ (4)

where ***y*** is the premium income, **X** is 17 original variables matrix, *X*_*j*_ = (*x*_1*j*_,…*x*_*nj*_)^*T*^, *j* = 1, 2,…,*p* are the predictors, ${\left|\beta \right|}^{2}=\sum_{j=1}^{p}\beta_{j}^{2}$ and ${\left|\beta \right|}_{1}=\sum_{j=1}^{p}\left|\beta_{j}\right|$. $\hat{\beta }$ is elastic net estimator.

The above procedure is regarded as the penalized least squares. The solution of $\hat{\beta }$ in Eq (4) is similar to find the optimal solution when *α* = *λ*_2_ / (*λ*_1_ + *λ*_2_).


$$\hat{\beta }=\arg\underset{\beta }{\min}{\left|y-X\beta \right|}^{2},\;\text{subject}\;\text{to}\;(1-\alpha ){\left|\beta \right|}_{1}+\alpha {\left|\beta \right|}^{2}\leq t\text{for}\;\text{some}\;\text{t}.$$ (5)

**Fig 4:**
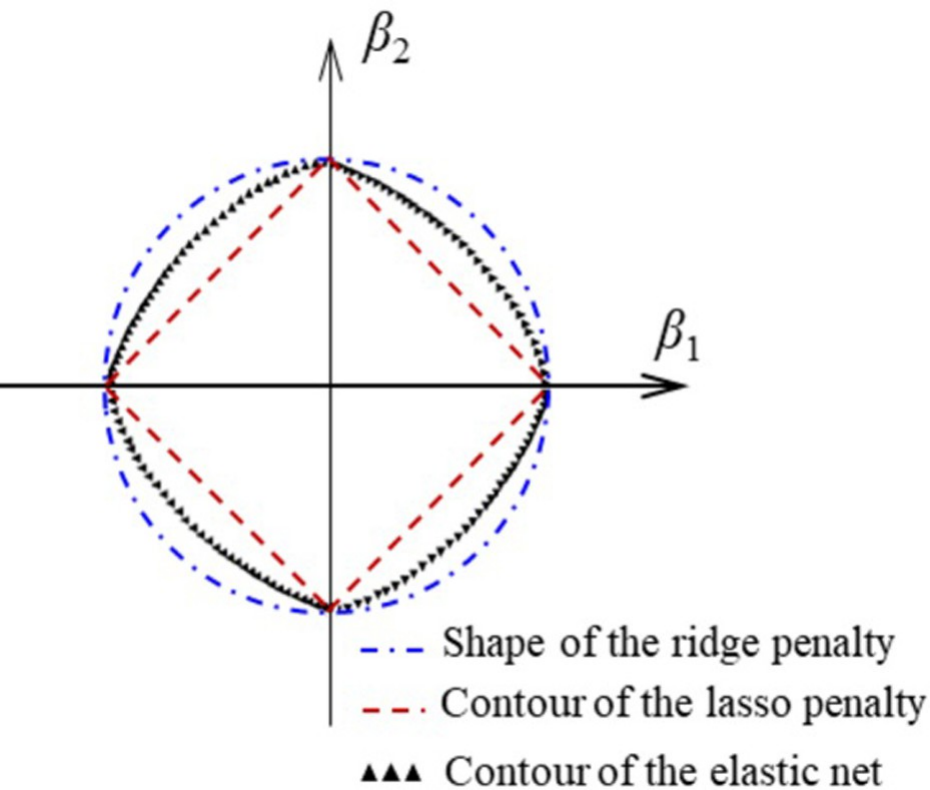
The comparison of ridge, lasso and elastic net.

The Eq (5) can be written as:

$$\hat{\beta }=\underset{\beta }{\arg\min}\left\{\sum_{i=1}^{N}(y_{i}-\beta_{0}-\sum_{j=1}^{p}x_{ij}\beta_{j}{)}^{2}+(1-\alpha )\sum_{j=1}^{p}\left|\beta_{j}\right|+\alpha \sum_{j=1}^{p}\beta_{j}{}^{2}\right\}$$ (6)

where $\sum_{i=1}^{N}(y_{i}-\beta_{0}-\sum_{j=1}^{p}x_{ij}\beta_{j}{)}^{2}$ is the sum of squares of the difference between the true value of premium income and the fitted value obtained from the characteristic variables (17 original variables including PM_2.5_, *etc*.). The function $(1-\alpha )\sum_{j=1}^{p}\left|\beta_{j}\right|+\alpha \sum_{j=1}^{p}\beta_{j}{}^{2}$is the elastic net penalty, the range of lasso penalty *α* is 0~1. Therefore, this method has the comprehensive features of lasso and ridge regression. A comparison of the three methods is shown in Fig 4.

**Fig 5:**
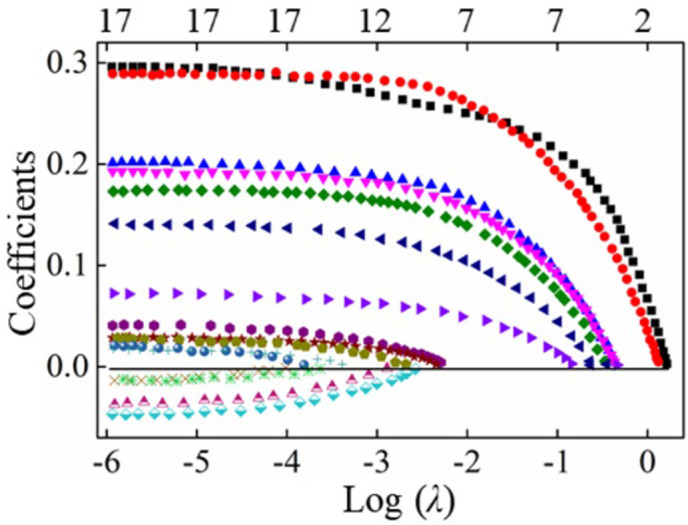
Coefficient solution path.

**Fig 6:**
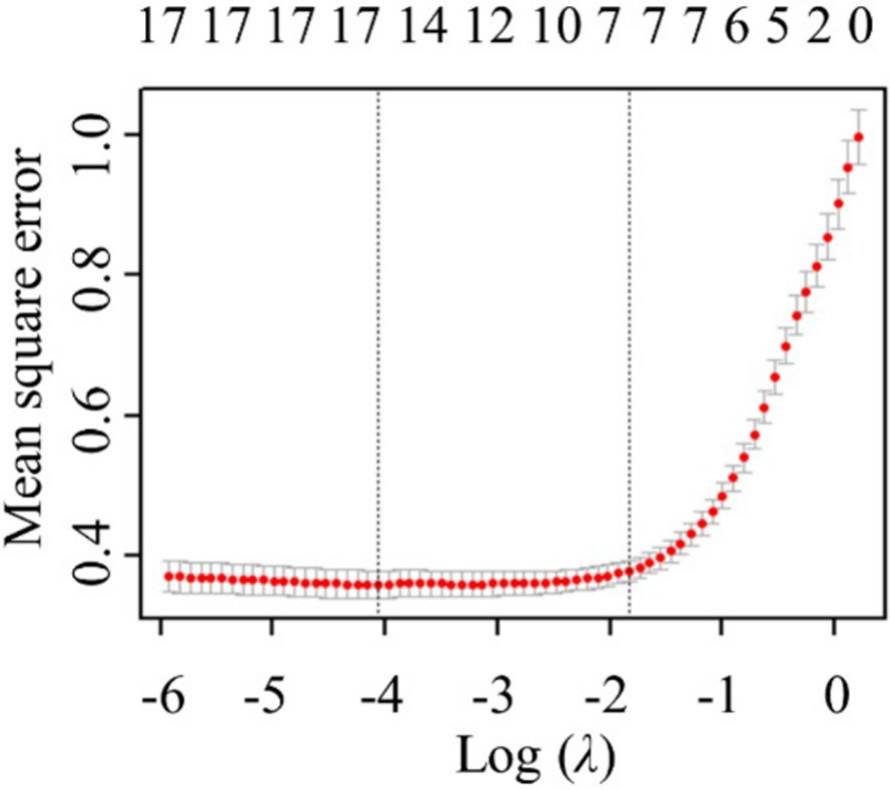
The corresponding trend of λ and the number of variables.

The results of the elastic net are shown in Figs 5 and 6. Finally, the 7 explanatory variables are obtained. Table 1 show the statistical characteristics values of the explained variables, core variables, control variables.

**Table 1:** Descriptive statistics of main variables.

Variable	Full name	samples	Mean	Standard deviation	Minimum	Maximum
Preincome	Premium income	2680	7359.83	13094.62	23.94	197315.3
PM_2.5_	The concentration of PM_2.5_	2680	37.42422	15.95459	4.6764	86.47989
Population	Population	2680	148.9809	185.7041	15.1	2465
Podensity	Population density	2680	916.1335	876.6028	12.4602	11449.3
Terproperty	The proportion of the added value of the tertiary industry in regional GDP	2680	44.60842	11.18169	9.76	85.95
Industry	Number of industrial enterprises	2680	596.3416	1078.555	6	8413
Sturatio	The proportion of college students in the population	2680	0.041	0.040	0.00000025	0.242
Sewage	Urban domestic sewage treatment rate	2680	81.87723	16.69396	0.16	100

Population (*million*). A larger population increases the possibility of purchasing insurance. Therefore, cities with larger populations will have higher premium income.

Population density (“Podensity”, 100 *people*/*km*^2^). “Podensity” represents the degree of population aggregation, and the larger the value, the more serious the haze pollution.

The ratio of the added value of the tertiary industry in the regional GDP (“Terproperty”, %). The insurance market belongs to a tertiary industry, and this indicator indicates the regional insurance development.

The number of industrial enterprises (referred to as "Industry”). The use of fossil and pollutants emissions in production activities of heavy industrial is the source of PM_2.5_, and the number of industrial enterprises can reflect the degree of the haze pollution [24].

The ratio of the population with higher education (referred to as “Sturatio,” unit: %). Relevant empirical researches verify that education level has a significant impact on family behavior in asset selection and commercial insurance purchases [25].

Urban domestic sewage treatment rate (referred to as “Sewage,” unit: %). There are many sources of PM_2.5_ in urban areas, including heat and electricity (32.83%) [26], and the energy consumption of wastewater treatment plants (WWTPs) in the worldwide accounts for 1% - 3% of the society’s total energy consumption [27].

### 2.3. Endogeneity and instrumental variables

**Fig 7:**
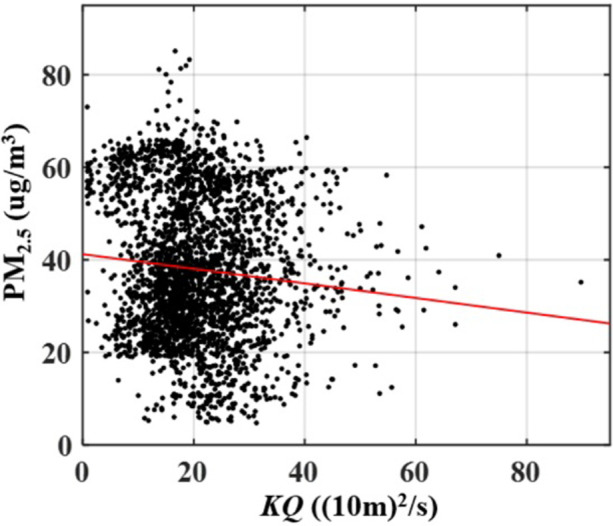
Scatter diagram and regression line of the correlation between PM_2.5_ and air flow coefficient (*KQ*).

Since haze pollution is spatially diffuse, its spillover can be evaluated by using instrumental variables (IV). The instrumental variables should be related to endogenous variable (PM_2.5_) and have no direct impact on the explained variable (premium income). This paper uses the ERA-INTERIM raster meteorological data [28, 29]. The atmospheric quantitate model is used to obtain the air flow coefficient (*KQ*), the definition of the *KQ* from the study [3]. *KQ* is adopted as an instrumental variable of haze pollution. Fig 7 exhibits the negative correlation between the *KQ* and PM_2.5_.

### 2.4. Regional governance, haze pollution reduction and insurance development

According to the methods of Chen et al. [30], the frequency and ratio of words related to environment in government reports are selected as an indicator of environmental governance in prefecture-level cities. The reasons for satisfying the exogenous assumption of IV are as follows. (a) The government reports are a summary of last year’s work and are released at the beginning of this year, which will not influence economic activities in this year and avoid the endogeneity issue. (b) The regional environmental governance used in this paper is from higher-level (provincial-level), the policies of lower-level (prefecture-level cities) governments are difficult to affect higher-level decisions, which will also alleviate endogeneity issue.

**Fig 8:**
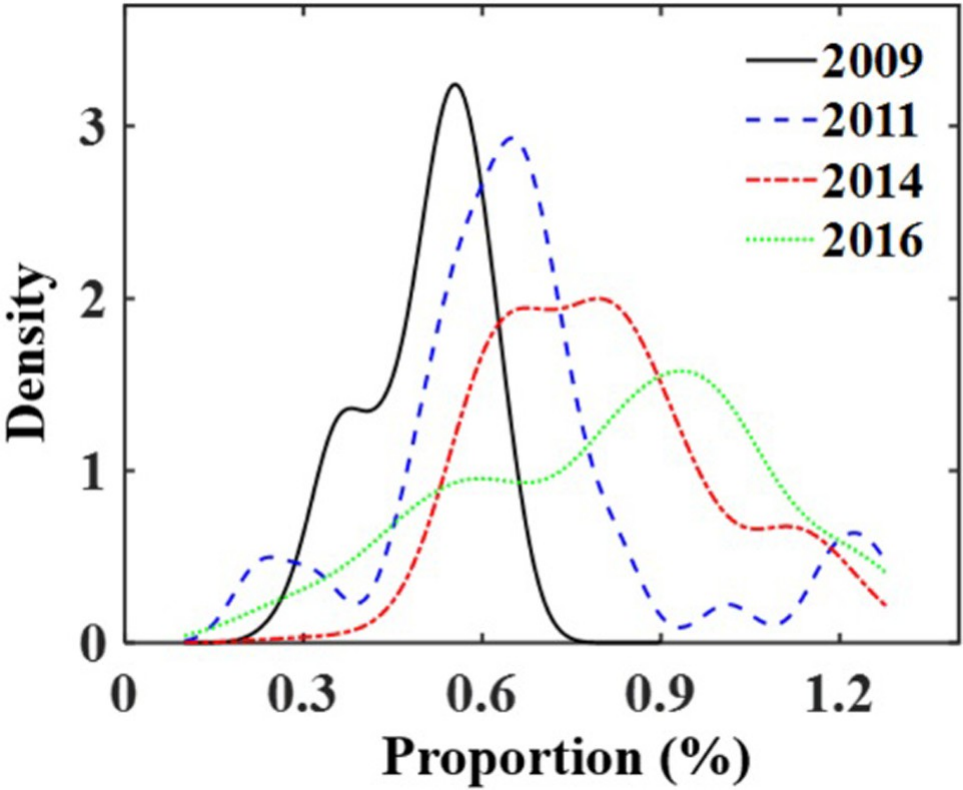
The proportion of environment-related words in the government work report.

The government work reports from 30 provinces during 2009–2018 were collected from official websites, the occurrence frequency of vocabulary words related to the environment were counted by using text segmentation [31]. The relevant words mainly included “*environmental protection*, *pollution*, *energy consumption*, *environmental improvement*, *COD (Chemical Oxygen Demand)*, *green*, *ecology*, *SO*_*2*_, *CO*_*2*_, *PM*_*2*.*5*_” etc. These words are widely used in government reports, which can indicate the government’s efforts in environmental protection. The nuclear density curve of environment-related words in the government reports is shown in Fig 8. It is found that the words of environment increases over time, which is consistent with the government’s increasing emphasis on environmental issues.

The provincial-level governance shows different effects on prefecture-level cities because of the difference of industry structure in each city. For example, the cities with a higher proportion of heavy industry, the regional environmental governance is stricter. To highlight the variability of regional environmental governance, similar to the study of Bartik [32], the ratio of secondary industry’s added value of cities in GDP is calculated. The statistical results of the words of environment information are shown in Table 2.

**Table 2:** Statistics on the frequency of environmental policy vocabulary of government work reports in 2018.

	PM2.5/ PM10	SO2	Atmosphere/air	low carbon/CO2	ecology	environmental protection/ environmental improvement	pollution	energy consumption	emission reduction	green	COD/sewage
**Anhui**	**1**	**0**	**2**	**1**	**30**	**10**	**16**	**2**	**2**	**24**	**2**
**Beijing**	0	0	9	2	29	6	24	2	0	9	5
**Chongqing**	1	0	4	0	32	15	16	2	1	9	2
**Fujian**	0	0	6	0	44	8	17	3	2	16	3
**Gansu**	0	0	5	1	52	12	9	1	1	20	1
**Guangdong**	0	0	5	1	26	8	24	2	2	9	4
**Guangxi**	0	0	4	2	43	11	8	2	2	5	2
**Guizhou**	0	0	1	0	38	8	8	0	4	27	7
**Hainan**	1	0	6	0	59	15	8	0	2	5	7
**Hebei**	3	0	5	1	23	11	18	3	2	11	6
**Heilongjiang**	2	1	3	1	24	3	5	2	0	9	3
**Henan**	6	0	8	0	26	6	23	5	2	10	2
**Hubei**	0	0	2	0	24	7	10	1	2	17	2
**Hunan**	0	0	0	0	15	7	10	1	2	11	5
**Jiangsu**	2	1	8	5	52	7	17	2	2	8	2
**Jiangxi**	0	0	2	2	49	4	4	2	2	27	1
**Jilin**	0	0	7	0	23	10	9	2	1	17	3
**Liaoning**	1	1	2	1	31	3	7	1	1	7	1
**Inner Mongolia**	0	0	2	1	23	5	13	1	2	12	0
**Ningxia**	0	0	1	0	36	12	7	1	2	10	4
**Qinghai**	0	0	1	2	51	7	4	0	2	30	1
**Shaanxi**	0	0	0	1	27	8	13	2	0	6	1
**Shandong**	2	0	5	0	26	9	11	3	1	8	0
**Shanghai**	0	0	6	1	24	7	10	4	0	10	4
**Shanxi**	2	0	6	3	29	8	15	2	1	7	2
**Sichuan**	5	0	2	0	32	9	15	1	1	15	3
**Tianjin**	2	0	2	1	23	7	12	1	2	21	7
**Xinjiang**	0	0	8	0	50	17	17	1	0	11	2
**Yunnan**	0	0	6	0	33	12	12	1	2	19	2
**Zhejiang**	4	0	1	0	21	5	8	3	1	10	6

Notes: the definition of the *COD* is same as the study [3]. *COD* represents the amount of oxygen required to oxidize the organic matter in water.

## 3. Discussion and analysis

### 3.1 Benchmark regression

Table 3 presents the benchmark regression results. From the first column, after controlling characteristic variables, haze pollution shows obvious negative impact on insurance development. Considering the impact of financial enterprises and heavy industrial enterprises on insurance development and environment pollution, the two factors are added to the benchmark regression. The second column shows the ratio of added value of tertiary industry to control the impact of tertiary industry development. A significant negative correlation between environment pollution and insurance development is also observed. In the third column, the number of industrial enterprises is included in the regression, which shows a significant negative correlation with insurance development. To investigate the time lag effect of haze pollution on insurance development, the lag of haze pollution is substituted into benchmark regression model.


$$Preincome_{i,j}=\alpha_{0}+\alpha_{1}PM_{2.5}{{}_{i,}}_{j-1}+\alpha_{2}X_{i,j}+\varepsilon_{i,j}$$ (7)

Thus, corresponding to columns 1–3, columns 4–6, the current PM_2.5_ lagged by one period, the negative effect of haze pollution on insurance development still exists, the results are significant. Control variable coefficients are as desired; cities with a larger population, higher education level, and less electricity consumption will have higher insurance premium income and more developed insurance market.

**Table 3:** Haze pollution and insurance development: Benchmark regression results.

	(1)	(2)	(3)	(4)	(5)	(6)	(7)	(8)	(9)
	current PM_2.5_			The lag of PM_2.5_			current PM_2.5_		
	Preincome	Preincome	Preincome	Preincome	Preincome	Preincome	Preincome	Preincome	Preincome
**PM**_**2.5**_	-0.078***	-0.077***	-0.071***				-0.094***	-0.100***	-0.099***
	(0.02)	(0.02)	(0.02)				(0.02)	(0.02)	(0.02)
**PM**_**2.5**_^**2**^							0.030***	0.029***	0.028***
							(0.01)	(0.01)	(0.01)
**L.PM**_**2.5**_				-0.049**	-0.048**	-0.050**			
				(0.02)	(0.02)	(0.02)			
**Industry**			-0.310***			-0.185***	-0.312***		
			(0.03)			(0.03)	(0.03)		
**Terproperty**		-0.010	-0.019		-0.014	-0.017	-0.018		-0.009
		(0.01)	(0.01)		(0.02)	(0.02)	(0.01)		(0.01)
**Population**	1.117***	1.114***	1.224***	1.088***	1.084***	1.164***	1.218***	1.111***	1.109***
	(0.04)	(0.04)	(0.04)	(0.04)	(0.04)	(0.05)	(0.04)	(0.04)	(0.04)
**Podensity**	0.013	0.014	0.007	0.011	0.011	0.008	0.010	0.016	0.016
	(0.01)	(0.01)	(0.01)	(0.02)	(0.02)	(0.02)	(0.01)	(0.01)	(0.01)
**Sturatio**	0.076***	0.077***	0.070***	0.072***	0.073***	0.071***	0.074***	0.079***	0.080***
	(0.02)	(0.02)	(0.02)	(0.02)	(0.02)	(0.02)	(0.02)	(0.02)	(0.02)
**Sewage**	-0.055***	-0.055***	-0.049***	-0.054***	-0.054***	-0.053***	-0.049***	-0.054***	-0.054***
	(0.01)	(0.01)	(0.01)	(0.01)	(0.01)	(0.01)	(0.01)	(0.01)	(0.01)
**Cons**	YES	YES	YES	YES	YES	YES	YES	YES	YES
**Year FEs**	YES	YES	YES	YES	YES	YES	YES	YES	YES
**City FEs**	YES	YES	YES	YES	YES	YES	YES	YES	YES
***R*** ^**2**^	0.887	0.887	0.892	0.898	0.898	0.899	0.892	0.887	0.887
**adj. *R*** ^**2**^	0.873	0.873	0.879	0.884	0.884	0.886	0.880	0.874	0.874
***N***	2680	2680	2680	2412	2412	2412	2680	2680	2680

Notes: Standard errors in parentheses.

* p < 0.10,

** p < 0.05,

*** p < 0.01; The number in brackets is the standard deviation, L. is a one-period lag operator. Same below.

The results of the benchmark regression indicate that haze pollution significantly inhibit insurance development in China, the impact coefficient is -0.071. The destructiveness of haze pollution largely depends on pollution severity, the more severe haze pollution is, and the more insurance can be sold [7]. Therefore, the relationship between haze pollution and insurance development may be different in different periods of haze severity. Considering the Environmental Kuznets Curve (EKC) effect, a quadratic term of PM_2.5_ concentration is added to regression model for checking the above-mentioned effect. From the results in columns 7–9 of Table 3, the first-order coefficient of PM_2.5_ is obviously negative, whereas the second-order coefficient is significant positive. Therefore, the relationship between haze pollution and insurance development does not satisfy traditional EKC hypothesis and demonstrates a "U"-shaped curve.

### 3.2 Analysis of impact mechanisms

The above results demonstrate that haze pollution show a remarkably adverse impact on Chinese insurance development. In this section, the underlying mechanisms of this relationship is analyzed in detail. Numerous researches have demonstrated that environment pollution can affect the decision-making of residents by influencing emotions [21, 22]. The insurance density is adopted to evaluate residents’ participation in insurance [33], which reflects the impacts of haze pollution on emotions. In addition, the haze pollution is spatially heterogeneous in China. Therefore, the samples can be divided into three parts, east west, and central of China, according to the geographical location, and subsample regression is adopted to analysis the underlying mechanisms.

Table 4 exhibits the corresponding results of the residents’ participation in insurance. The coefficients of insurance density in columns 1–2 are significantly positive, which indicates that active residents’ participation in insurance will promote the development of insurance. However, due to haze pollution influences people’s emotions and alters residents’ decision-making [34]. The coefficients in columns 3–7 are significantly negative, which indicates that haze pollution decreases residents’ participation. To alleviate possible endogeneity problem, columns 8–10 in Table 4 adopts the lagging terms of haze pollution to investigate the effect of haze pollution on insurance density. The negative impact in eastern region is higher than the impact in western and central regions, which is consistent with the fact that residents in developed areas- eastern region are more sensitive to haze pollution. The definition of eastern, middle, and western regions of China is followed the code issued by the National Bureau of Statistics in 2003 according to economic level and geographical location.

**Table 4:** Haze pollution and insurance development: Analysis of residents’ participation in the insurance.

	Insurance density and premium income		The impact of PM_2.5_ on insurance density							
			All samples		East	West	Central	East	West	Central
	(1)	(2)	(3)	(4)	(5)	(6)	(7)	(8)	(9)	(10)
	Preincome	Preincome	Insdensity	Insdensity	Insdensity	Insdensity	Insdensity	Insdensity	Insdensity	Insdensity
**Insdensity**	0.171***									
	(0.02)									
**L.Insdensity**		0.161***								
		(0.02)								
**PM**_**2.5**_			-0.180***		-0.642***	-0.307***	-0.161***			
			(0.05)		(0.09)	(0.10)	(0.02)			
**L.PM**_**2.5**_				-0.192***				-0.410***	-0.406***	-0.144***
				(0.02)				(0.10)	(0.08)	(0.03)
**Population**	0.897***	0.897***	0.633***	0.080	1.717***	-0.047	0.208***	1.820***	-0.017	0.222***
	(0.03)	(0.04)	(0.11)	(0.05)	(0.21)	(0.06)	(0.08)	(0.23)	(0.06)	(0.08)
**Podensity**	0.006	0.011	0.004	0.015	0.054	0.050	0.032	0.100**	0.070	0.025
	(0.01)	(0.01)	(0.02)	(0.02)	(0.05)	(0.06)	(0.02)	(0.05)	(0.06)	(0.02)
**Terproperty**	-0.022**	-0.014	-0.035	0.149***	0.021	0.085**	0.127***	0.014	0.076*	0.126***
	(0.01)	(0.01)	(0.03)	(0.02)	(0.06)	(0.04)	(0.02)	(0.07)	(0.04)	(0.02)
**Industry**	-0.187***	-0.126***	-0.215***	0.311***	-0.275***	0.213	0.606***	-0.216	0.171	0.620***
	(0.02)	(0.03)	(0.07)	(0.04)	(0.10)	(0.14)	(0.18)	(0.13)	(0.14)	(0.19)
**Sturatio**	0.061***	0.035**	-0.021	0.013	0.137*	0.060	-0.051**	0.166**	0.065*	-0.046**
	(0.01)	(0.02)	(0.04)	(0.02)	(0.07)	(0.04)	(0.02)	(0.08)	(0.04)	(0.02)
**Sewage**	-0.001	-0.014	-0.111***	0.113***	-0.161***	0.125***	0.124***	-0.188***	0.122***	0.109***
	(0.01)	(0.01)	(0.03)	(0.02)	(0.04)	(0.03)	(0.03)	(0.06)	(0.04)	(0.04)
**Cons**	YES	YES	YES	YES	YES	YES	YES	YES	YES	YES
**Year FEs**	YES	YES	YES	YES	YES	YES	YES	YES	YES	YES
**City FEs**	YES	YES	YES	YES	YES	YES	YES	YES	YES	YES
***R*** ^**2**^	0.905	0.910	0.536	0.547	0.561	0.475	0.477	0.552	0.488	0.502
**adj. *R*** ^**2**^	0.894	0.898	0.481	0.487	0.504	0.402	0.409	0.486	0.410	0.430
***N***	2680	2412	2680	2412	950	720	1010	855	648	909

Notes: Standard errors in parentheses.

* p < 0.10,

** p < 0.05,

*** p < 0.01; the number in brackets is the standard deviation, L. is a one-period lag operator. Same below.

The eastern region includes Beijing, Tianjin, Hebei, Liaoning, Shanghai, Jiangsu, Zhejiang, Fujian, Shandong, Guangdong, Guangxi and Hainan. The central region includes Shanxi, Inner Mongolia, Jilin, Heilongjiang, Anhui, Jiangxi, Henan, Hubei and Hunan. The western regions include Sichuan, Guizhou, Yunnan, Tibet, Shaanxi, Gansu, Ningxia, Qinghai, Xinjiang, and Guangxi. This article divides the 268 prefecture-level cities into the corresponding regions.

GDP is widely recognized as the best indicator to measure regional economic development, the relationship between the haze pollution and insurance development is indirect, and the bridge is economic development (GDP). Haze pollution can hindered economic development, which will further affects the residents’ income [11] and household budgets for insurance purchase [12]. Therefore, we can use GDP as the bridge to examine the impact of haze pollution on the development of insurance. Table 5 shows that the impact of GDP on insurance development is positive with the coefficient of 1.229, which indicates that a high level of economic development drives the development of regional insurance, the results is similar to the study of Outreville [35]. At the same time, haze pollution significantly inhibits economic development with an impact coefficient of -0.065 (Table 5), which is consistent with previous literatures [9, 10]. In addition, economic development is positively correlated with residents’ income [11]. Haze pollution hinders economic development, which will reduce residents’ income and household budgets [12]. Therefore, haze pollution hindering economic development will reduce household budgets, which will decrease insurance purchase demand [36] and hinder insurance development. In addition, the space heterogeneity of the influence of haze pollution on economic development is also observed, the difference in eastern region is higher than others regions, the similar result is reported by Nie et al. [37].

**Table 5:** Haze pollution and insurance development: The level of economic development.

	GDP and premium income		The impact of PM_2.5_ on GDP							
			All samples		East	West	Central	East	West	Central
	(1)	(2)	(3)	(4)	(5)	(6)	(7)	(8)	(9)	(10)
	Preincome	Preincome	GDP	GDP	GDP	GDP	GDP	GDP	GDP	GDP
**GDP**	1.229***									
	(0.06)									
**L. GDP**		1.283***								
		(0.07)								
**PM**_**2.5**_			-0.065***		-0.117***	-0.052***	-0.037***			
			(0.01)		(0.04)	(0.02)	(0.01)			
**L.PM**_**2.5**_				-0.064***				-0.118**	-0.042**	-0.032*
				(0.02)				(0.05)	(0.02)	(0.02)
**Population**	-0.321***	-0.233**	1.255***	1.161***	1.885***	0.982***	0.728***	1.765***	0.943***	0.508*
	(0.09)	(0.09)	(0.09)	(0.09)	(0.17)	(0.07)	(0.24)	(0.18)	(0.08)	(0.26)
**Podensity**	-0.032***	-0.038***	0.032**	0.035**	0.118***	0.014	-0.050**	0.131***	0.013	-0.064**
	(0.01)	(0.01)	(0.01)	(0.01)	(0.03)	(0.01)	(0.02)	(0.03)	(0.01)	(0.03)
**Terproperty**	-0.009	-0.009	-0.008	-0.009	0.010	-0.008	-0.022*	0.010	-0.005	-0.019
	(0.01)	(0.01)	(0.01)	(0.01)	(0.03)	(0.01)	(0.01)	(0.03)	(0.01)	(0.01)
**Industry**	0.025	0.031	-0.272***	-0.137*	-0.335***	-0.049	0.079	-0.189**	-0.029	0.415
	(0.04)	(0.05)	(0.07)	(0.08)	(0.07)	(0.05)	(0.30)	(0.10)	(0.06)	(0.40)
**Sturatio**	-0.021*	-0.027**	0.074***	0.069***	0.172***	0.050***	0.014	0.164***	0.037**	0.007
	(0.01)	(0.01)	(0.02)	(0.02)	(0.03)	(0.02)	(0.03)	(0.03)	(0.02)	(0.02)
**Sewage**	-0.011**	-0.012**	-0.031***	-0.031***	-0.020	-0.021***	-0.027***	-0.012	-0.025***	-0.026**
	(0.00)	(0.01)	(0.01)	(0.01)	(0.02)	(0.01)	(0.01)	(0.03)	(0.01)	(0.01)
**Cons**	YES	YES	YES	YES	YES	YES	YES	YES	YES	YES
**Year FEs**	YES	YES	YES	YES	YES	YES	YES	YES	YES	YES
**City FEs**	YES	YES	YES	YES	YES	YES	YES	YES	YES	YES
***R*** ^**2**^	0.962	0.964	0.954	0.961	0.960	0.975	0.919	0.966	0.980	0.935
**adj. *R*** ^**2**^	0.958	0.959	0.948	0.956	0.955	0.971	0.908	0.961	0.976	0.926
***N***	2680	2412	2680	2412	950	720	1010	855	648	909

Notes: Standard errors in parentheses,

* p < 0.10,

** p < 0.05,

*** p < 0.01; The number in brackets is the standard deviation, L. It is a one-period lag operator. Same below.

## 4. Regional governance and insurance development: Instrumental variables regression

### 4.1. Results of the instrumental variables regression

The air flow coefficient and regional environmental governance are selected as instrumental variables (IV) to mitigate endogeneity. The using of other function of *KQ* is to control spillover effect of haze pollution. IV regression is used to alleviate the potential endogeneity of benchmark regression, and to evaluate the effect of regional governance on insurance development. According to the first-stage regression, the impact of regional governance can be identified.

The frequency and ratio of environmental vocabularies in the government reports and the proportion of added -value of the secondary industry in regional GDP are regarded as environmental governance variables, and their interactions are explored. As shown in Table 6, the environmental governance variables in the first-stage regression not only alleviate endogeneity, but also examine the regional governance effects within a unified framework. The first-stage regression results indicate that no matter what criterion is selected, environmental governance can obviously reduce the environment pollution under a significance level of 1% (Table 6).

**Table 6:** Regional governance and insurance development: Instrumental variables regression.

	(1)	(2)	(3)	(4)
**First-stage**	**PM**_**2.5**_			
**Frequency**	-0.074***			
	(0.010)			
**Percentage**		-0.061***		
		(0.010)		
Frequency*Secondary			-0.090***	
			(0.024)	
Percentage*Secondary				-0.054***
				(0.016)
**KQ**	-0.083***	-0.080***	-0.078***	-0.079***
	(0.011)	(0.011)	(0.012)	(0.012)
***F***	111.26	107.19	108.72	108.07
**Second-stage**	Preincome			
**PM**_**2.5**_	-0.216 ***	-0.190***	-0.264***	-0.180***
	(0.061)	(0.061)	(0.069)	(0.067)
**Cons**	YES	YES	YES	YES
**Controls**	YES	YES	YES	YES
**Year FEs**	YES	YES	YES	YES
**City FEs**	YES	YES	YES	YES
***R*** ^**2**^	0.8333	0.8323	0.8304	0.8302
**adj. *R*** ^**2**^	0.8135	0.8124	0.8102	0.8100
***N***	2680	2680	2680	2680

Notes: Standard errors in parentheses.

* p < 0.10,

** p < 0.05,

*** p < 0.01; The number in brackets is the standard deviation, L. is a one-period lag operator. Same below.

The first stage *F* is larger than 10, thus, the "weak IV" are eliminated. This result is similar to benchmark regression results reported in Table 3, which also indicates that haze pollution has an unfavorable effect on Chinese insurance development. The absolute value of coefficient of PM_2.5_ is larger than the value in the benchmark regression, which underestimate the negative effects because of potential endogeneity. Specifically, the first column of Table 6 demonstrates that one environment-related word increase in the government work reports, the concentration of PM_2.5_ decreases 0.074 *μg/m*^*3*^. Correspondingly, the second-stage results show that one increase in PM_2.5_ (1*μg/m*^*3*^), premium income decreases by 2.16 million CNY. Combining the coefficients of the first and second stage in the first column, it can be seen that one environment-related word increase in government reports, the premium income will increase 1598.4 CNY. To control the report’s length, the proportion of environmental words is selected to represent the environmental governance in the second column. The results show that if the proportion increases by 0.1%, PM_2.5_ decreases to 0.61 *μg*/*m*^*3*^, and premium income increases by 1159 CNY. The environment-related vocabularies generally account for only 0.73% in the full text, 0.1% increase in frequency is considerable. The results in columns 3–4 are negative under 1% significance level, which indicates that cities with a larger proportion of added value of secondary industry in regional GDP are more sensitive to environmental governance. Based on the above results, haze pollution significantly hinders insurance development. The government is indispensable in the process of haze governance and insurance development.

### 4.2. Robustness analysis

The robustness tests were conducted based on the results reported in column 1 in Table 6 to examine the results reliability. The results of robustness tests are showed in Table 7. In order to make the sample more homogenous, the sample of the municipality is removed due to administrative autonomy and uniqueness. The results in the first column of Table 7 are consistent with the baseline. Furthermore, regression results in columns 2–3 of Table 7 are the same as above-mentioned when highest, lowest concentrations of 5% and 1% of PM_2.5_ are excluded. In addition, PM_2.5_ is related to SO_2_ emissions and dust emissions. The pollution sources have a possible remarkable impact on economic and insurance development, which will lead to biases in the omitted variables. Therefore, SO_2_ and dust emissions are added to well identify haze pollution’s impact on Chinese insurance development. Compared to the results in first column of Table 6 with that in fourth column of Table 7, it is found that although the absolute value of PM_2.5_ coefficient is decreased, the significance did not change. The urban household’s life are also one of the sources of direct PM_2.5_ pollutants [2, 26]. However, the components are exogenous, which have no obvious effect on residents’ willingness to purchase insurance. Therefore, the fifth column of Table 7 uses per capita gas as an additional IV for haze pollution. The results indicate that coefficient and significance of environmental governance are in line with baseline. The coefficient of per capita gas usage is significantly positive and is same as expectation. Although the significance of PM_2.5_ has decreased, the relationship is still significant, which certifies the robustness of this paper. In addition, the control variables might exist endogeneity issue because of "reverse causality", which lagged one period in the sixth column of Table 7. Although the significance decreased, the causality remains unchanged.

**Table 7:** Regional governance and insurance development: Robustness analysis.

	(1)	(2)	(3)	(4)	(5)	(6)
**First-stage**	**PM**_**2.5**_					
**Frequency**	-0.074***	-0.036***	-0.035***	-0.043***	-0.075***	-0.070***
	(0.010)	(0.009)	(0.009)	(0.009)	(0.010)	(0.011)
**KQ**	-0.082***	-0.036***	-0.030**	-0.085***	-0.083***	-0.095***
	(0.011)	(0.012)	(0.013)	(0.009)	(0.011)	(0.010)
**Gas**					0.031**	
					(0.013)	
**Sulfur**				-0.074***		
				(0.021)		
**Dust**				0.008		
				(0.009)		
***F***	111.72	898.39	864.64	828.69	111.82	94.08
**Second-stage**	**Preincome**					
**PM**_**2.5**_	-0.206***	-0.564***	-0.544**	-0.235***	-0.163**	-0.090*
	(0.058)	(0.020)	(0.231)	(0.076)	(0.064)	(0.060)
**Cons**	YES	YES	YES	YES	YES	YES
**Controls**	YES	YES	YES	YES	YES	YES
**Year FEs**	YES	YES	YES	YES	YES	YES
**City FEs**	YES	YES	YES	YES	YES	YES
***R*** ^**2**^	0.8325	0.8177	0.8132	0.8309	0.8337	0.8274
**adj. *R*** ^**2**^	0.8126	0.7901	0.7844	0.8082	0.8139	0.8044
***N***	2650	2128	2086	2412	2680	2412

Notes: Standard errors in parentheses.

* p < 0.10,

** p < 0.05,

*** p < 0.01; The number in brackets is the standard deviation, L. is a one-period lag operator. Same below.

## 5. Conclusion and policy implications

This paper investigates the haze pollution’s effect on insurance development, evaluates haze reduction effect by regional environmental governance and its impact on the development of insurance in China. Through the results of fixed effects, the haze pollution has an unfavorable influence on insurance development. Haze pollution can suppress the insurance development through the two important underlying mechanisms, residents’ emotions and economic development. These two negative effects exhibit regional variations, which gradually weaken from the eastern, western regions, towards Chinese central region. In addition, the air flow coefficient and government environmental regulations are adopted to investigate the reliability of results as instrumental variables of PM_2.5_ concentrations. The conclusion is that the Chinese government’s environmental governance has effectively reduced haze pollution and its negative impact on the insurance development.

The relationship between economic development and haze pollution is mutually restrictive. The governments should establish standards for regional haze pollution governance, strengthen collaborative governance on haze pollution in various regions to address haze pollution spillover effects, crack such a vicious circle and dilemma. For insurance, the government should provide policy encouragement to stimulate residents’ enthusiasm. The local insurance companies need to adjust insurance premium rates according to the heterogeneity of regional haze pollution. In this way, the win-win goal that haze pollution is reduced and the social security of insurance is better implemented will be achieved.

## Supporting information

S1 Dataset PM_2.5_ concentration of China in 2009 and 2018. (XLSX)

S2 Dataset Insurance density of China in 2009 and 2018. (XLSX)

S3 Dataset Data of correlation between PM_2.5_ and air flow coefficient (KQ) corresponding to Fig 7. (XLSX)
